# Prediction of the Damage-Associated Non-Synonymous Single Nucleotide Polymorphisms in the Human *MC1R* Gene

**DOI:** 10.1371/journal.pone.0121812

**Published:** 2015-03-20

**Authors:** Diego Hepp, Gislene Lopes Gonçalves, Thales Renato Ochotorena de Freitas

**Affiliations:** 1 Departamento de Genética, Instituto de Biociências, Universidade Federal do Rio Grande do Sul, Porto Alegre, Rio Grande do Sul, Brazil; 2 Instituto de Alta Investigación, Universidad de Tarapacá, Antofagasta, 1520 Arica, Chile; 3 Instituto Federal de Educação, Ciência e Tecnologia do Rio Grande do Sul—Câmpus Porto Alegre, Rio Grande do Sul, Brazil; University of Texas, UNITED STATES

## Abstract

The melanocortin 1 receptor (*MC1R*) is involved in the control of melanogenesis. Polymorphisms in this gene have been associated with variation in skin and hair color and with elevated risk for the development of melanoma. Here we used 11 computational tools based on different approaches to predict the damage-associated non-synonymous single nucleotide polymorphisms (nsSNPs) in the coding region of the human *MC1R* gene. Among the 92 nsSNPs arranged according to the predictions 62% were classified as damaging in more than five tools. The classification was significantly correlated with the scores of two consensus programs. Alleles associated with the red hair color (RHC) phenotype and with the risk of melanoma were examined. The R variants D84E, R142H, R151C, I155T, R160W and D294H were classified as damaging by the majority of the tools while the r variants V60L, V92M and R163Q have been predicted as neutral in most of the programs The combination of the prediction tools results in 14 nsSNPs indicated as the most damaging mutations in *MC1R* (L48P, R67W, H70Y, P72L, S83P, R151H, S172I, L206P, T242I, G255R, P256S, C273Y, C289R and R306H); C273Y showed to be highly damaging in SIFT, Polyphen-2, MutPred, PANTHER and PROVEAN scores. The computational analysis proved capable of identifying the potentially damaging nsSNPs in *MC1R*, which are candidates for further laboratory studies of the functional and pharmacological significance of the alterations in the receptor and the phenotypic outcomes.

## Introduction

The melanocortin 1 receptor (*MC1R*) gene encodes for a G protein-coupled receptor (GPCR) with seven transmembrane domains involved in the control of melanogenesis. Ligation of the α-melanocyte stimulating hormone (α-MSH) to MC1R stimulates adenylate cyclase, with a consequent increase of cAMP levels that leads to the activation of tyrosinase (TYR) and other enzymes, resulting in the switch from the synthesis of phaeomelanin (red/yellow pigment) to eumelanin (black/brown pigment) in melanocytes [1].

The human MC1R protein contains 317 amino acids encoded in a single exon, and shows many polymorphisms that have been described in different populations [2]. Some human *MC1R* variants have been associated with variation in hair and skin pigmentation and with increased risk of developing melanoma and other skin cancers, and have been characterized in laboratory studies [3] [4] [5] [6] [7] [8] [9]. However, many of the polymorphisms have unknown effects. The non-synonymous single nucleotide polymorphisms (nsSNPs) in the coding region alter the corresponding proteins. These changes may affect the protein functions in many different ways, for instance by altering the catalytic or ligand binding sites, leading to improper protein folding, incorrect intracellular transportation, or decrease in the stability or loss of function of the gene product [10] [11] [12] [13] [14] [15] [16] [17] [18]. Understanding which molecular variations are related to Mendelian or complex diseases and to variations in phenotype is a challenge in genetic research [19]. Genome-wide association studies (GWAS) are powerful approaches to detect complex disease associated SNPs [20] [21] [22] [23] [24] however, factors as the degree of linkage disequilibrium between the disease variant and the SNP marker, difference in allele frequencies and the choose of the SNPs affect GWAS studies, resulting in lower detection power and in the demand of much larger samples than association studies using targeted candidate loci [25] [26] [27]. While *in vitro* tests can assess the effect of specific variations, it is laborious and time-consuming to evaluate the large amount of variation in the human genome [28].

Determining which SNPs affect the phenotype would make it possible to identify the molecular mechanisms of disease and phenotypic variation, and to help select the most important for association studies with populations. Several tools have been developed to differentiate the deleterious or disease-associated SNPs occurring in a gene from the neutral or tolerated alterations, and these tools use approaches based on different features [10]. These approaches include sequence-based methods that use evolutionary information on the amino-acid conservation in the gene, based on multiple sequence alignment (MSA) of homologous proteins in related species. Assuming that amino acids that are highly important for the structure and function of the protein will be more conserved in a protein family, mutations in those positions are more likely to be deleterious. Methods based on the structural, physical and chemical properties of the wild and mutant proteins also are available, and allow the identification of the SNPs that affect the stability and function of the protein [29] [30]. Other tools use machine-learning methods (such as the support vector machine, SVM; or Random Forest, RF) to predict the association of the SNPs with disease. These tools combine properties of the amino acid residues, structural information and evolutionary conservation, and databases that contain validated information about the biochemical and clinical evidence for SNPs known to be deleterious [19] [28]. In order to combine the results of the various tools, consensus predictors have been developed to allow comparison between methods that use different analytical approaches [10] [31]. Studies using combination of different prediction tools have identified deleterious mutations in genes involved in different biological processes, including, for example, cancer (breast cancer 1, early onset—*BRCA1* gene) [32], *STIL* gene [33], Centromere-associated protein-E gene (CENP-E) [34], leukemia (c-abl oncogene 1—*ABL1* gene) [35], lipoprotein metabolism (ATP-binding cassette transporter A1—*ABCA1* gene) [36], cardiomyopathy (beta myosin heavy chain—*MyH7* gene) [28], oxidative stress (superoxide dismutase 2—*SOD2* gene) [37], amyotrophic lateral sclerosis (superoxide dismutase 1—*SOD1* gene) [38], and melanogenesis (receptor tyrosine kinase—*KIT* gene [39], oculocutaneous albinism type 2—*OCA2*—P protein gene [40], tyrosinase—*TYR* gene [41], and tyrosinase-related protein 1—*TYRP1* gene [42]), resulting in the establishment of the mutations with the highest pathogenic prediction.

Here we used prediction tools to evaluate 92 nsSNPs in the *MC1R* gene in relation to their damaging or pathogenic effects, and to predict the disease-associated variation.

Thus, by the combination of the prediction tools we classified the nsSNPs in the *MC1R* gene, and selected those that are the most likely to affect the function of the receptor in a way that could result in disease or phenotypic variation in pigmentation.

## Material and Methods

### Data

Human *MC1R* gene data were obtained from OMIM (#155555 - http://www.ncbi.nlm.nih.gov/omim) and Entrez on the National Center for Biotechnology Information (NCBI) website, including Protein accession number (NP_002377) and mRNA accession number (NM_002386). The Uniprot accession number (Q01726) was obtained in the Swissprot database (http://expasy.org). The information on 92 SNPs in human *MC1R* was collected from dbSNP (http://www.ncbi.nlm.nih.gov/snp) including SNP ID (S1 Table), chromosome position, alleles and functional consequences, when available.

### Functional analysis Prediction

The nsSNPs were analyzed using 11 prediction tools: SIFT, MutPred, Polyphen-2, PROVEAN, I-Mutant 3.0, PANTHER, SNPs3D, Mutation Assessor, PhD-SNP, SNPs&GO and SNAP (Table 1) and the consensus prediction tools PON-P and PredictSNP 1.0. The data for chromosome location, amino acid sequence of the human *MC1R* gene (ref. Seq. NP_002377), Uniprot accession number (Q01726), position in the protein, and wild and mutated residue of the nsSNPs were used according to the program requirements. The prediction tools were selected by use different approaches in order to obtain a classification of the nsSNPs according to one or more features. The tools are freely accessible and described in the literature. Each program's approach is detailed below.

**Table 1:** Prediction tools used in the analysis.

Prediction tool	URL	Type	Reference
I-Mutant 3.0	http://gpcr2.biocomp.unibo.it/cgi/predictors/I-Mutant3.0/I-Mutant3.0.cgi	machine learning method (SVM)	30
Mutation Assessor	http://mutationassessor.org/	evolutionary conservation-based	34
MutPred	http://mutpred.mutdb.org/	evolutionary conservation and structure-based	26
PANTHER	http://www.pantherdb.org/tools/csnpScore.do	evolutionary conservation-based	31
PhD-SNP	http://snps.biofold.org/phd-snp/phd-snp.html	machine learning method (SVM)	35
PolyPhen-2	http://genetics.bwh.harvard.edu/pph2/index.shtml	evolutionary conservation and structure-based	27
PROVEAN	http://provean.jcvi.org/	evolutionary conservation-based	28
SIFT	http://sift.jcvi.org/	evolutionary conservation-based	25
SNAP	https://www.rostlab.org/services/SNAP/	machine learning method (neural-network), protein sequence and structure-based	37
SNPs&GO	http://snps-and-go.biocomp.unibo.it/snps-and-go/	machine learning method (SVM)	36
SNPs3D	http://snps3d.org/	machine learning method (SVM)	33
PON-P	http://bioinf.uta.fi/PON-P/	Consensus tool	38
PredictSNP 1.0	http://loschmidt.chemi.muni.cz/predictsnp	Consensus tool	15

The SIFT (Sorting Intolerant From Tolerant) tool uses a sequence homology based on the multiple sequence alignment (MSA) conservation approach to classify the nsSNPs as tolerated by or damaging to the protein. The SIFT score is the normalized probability that the amino acid change is tolerated. The score ranges from 0 to 1 with a cut-off score of 0.05. Amino acids substitutions with less than 0.05 are predicted to be deleterious, and those greater than or equal to 0.05 are predicted to be tolerated [43].

The MutPred tool was developed to classify an amino acid substitution as deleterious/disease-associated or neutral, based on three classes of attributes, the evolutionary conservation of the protein sequence, the protein structure and dynamics, and in functional properties, including secondary structure, solvent accessibility, stability, intrinsic disorder, B-factor, transmembrane helix, catalytic residues and others. It determines the changes at atomic and molecular level induced by the amino acid substitution. MutPred uses the RF (Random Forest) classifier to provide the *g* score for the prediction of the probability that the substitution is deleterious, and the *p* score for the indication of the structural and functional properties impacted, for instance, gain of helical propensity or loss of a phosphorylation site [44].

Polyphen-2 (Polymorphism Phenotyping v2) is a sequence and structure-based method that determines the structural and functional consequences of nsSNPs. The PolyPhen-2 calculates the posterior probability that a nsSNP is damaging by a Bayesian classifier [45]. The conservation of a position in the MSA and the deleterious effect on the protein structure results in the Position-Specific Independent Count (PSIC) score that ranges from 0 to 1. The classification of the nsSNPs results in Possibly Damaging and Probably Damaging (PSIC > 0.5) or Benign (PSIC < 0.5).

PROVEAN (Protein Variation Effect Analyzer) measures the damaging effect of variations in protein sequences [46]. The prediction is based on the change, caused by an nsSNP, in the similarity of the sequence to related protein sequences in a MSA. PROVEAN uses a delta alignment score based on the reference and variant versions of the protein sequence with respect to the alignment of homologous sequences [47]. A score equal or below the threshold of-2.5 determines the classification as a deleterious nsSNP.

I-Mutant 3.0 is a support vector machine (SVM) tool for the prediction of protein stability free-energy change (ΔΔG or DDG) on a specific nsSNP. It predicts the free energy changes starting from either the protein structure or the protein sequence [48]. A negative DDG value means that the mutation decreases the stability of the protein, while a positive DDG value indicates an increase in stability. I-Mutant 3.0 also implements a prediction of disease-associated SNPs from a sequence analysis based on a decision tree with the SVM-based classifier (SVM-Sequence) coupled to the SVM-Profile trained on sequence profile information. The nsSNPs are then classified as disease-related or neutral polymorphisms.

PANTHER (Protein ANalysis THrough Evolutionary Relationships) estimates the likelihood that a particular nsSNP will result in a functional alteration of the protein. It calculates the subPSEC (substitution position-specific evolutionary conservation) score based on a hidden Markov model alignment of evolutionarily related proteins [49] [50]. Substitution with subPSEC = 0 is indicated as functionally neutral, whereas negative values of subPSEC predict deleterious substitutions. A subPSEC score cut-off of-3 corresponds to a 50% probability that an nsSNP is deleterious to the protein, with a probability of causing a deleterious effect on the protein function (Pdeleterious) of 0.5.

SNPs3D analyzes the likely impact of nsSNPs on protein function by two methods, one based on the protein structure and stability, stemming from the hypothesis that many disease nsSNPs affect protein function primarily by decreasing protein stability. The program is intended to identify which amino acid substitutions significantly destabilize the folded state. The second model was based on analysis of homology in a sequence of families related to human proteins, through analysis of amino acid conservation at the affected sequence position [30] [51]. A positive SVM score indicates a variant classified as non-deleterious, and a negative score indicates a deleterious variant. The larger the score, the more confident is the classification of the nsSNP, with accuracy significantly higher for scores greater 0.5 or less than-0.5 [51].

The Mutation Assessor predicts the functional impact of amino acid substitutions in proteins based on evolutionary conservation of the affected amino acid in protein homologs, providing a rough estimate of the probability that the mutation has a phenotypic consequence at the level of the organism. It uses information based on the analysis of evolutionary conservation patterns in protein family multiple-sequence alignments, which are subject to selective forces at the level of the ability of the organism to survive and reproduce [52]. The analysis results in a functional impact score based on evolutionary information (FIS) that classifies the nsSNP as neutral, low, medium or high.

PhD-SNP (Predictor of Human Deleterious Single Nucleotide Polymorphisms) is a SVM-based classifier that uses protein sequence information to predict whether an nsSNP is disease-associated, based on a supervised training algorithm. The output is obtained from the frequencies of the wild and mutant residues, the number of aligned sequences, and the conservation index calculated for the position involved, and provides a prediction of disease-related (disease) or neutral polymorphism [53].

SNPs&GO is a method based on SVM to predict disease-related mutations from the protein sequence, that uses information derived from evolutionary information, protein sequence and function as encoded in the Gene Ontology (GO) terms annotation to predict if a given mutation can be classified as disease-related or neutral [54].

SNAP (Screening for Non-Acceptable Polymorphisms) is a neural network-based method for the prediction of the functional effects of nsSNPs. SNAP uses evolutionary information for the residue conservation within sequence families, aspects of protein structure, and annotations, when available. The SNAP network takes protein sequences and lists of mutants and provides a score for each substitution, which can then be translated into binary predictions of a neutral or non-neutral effect [55].

We compared the prediction results of our combined analysis with two consensus tools, PON-P and PredictSNP1.0. The PON-P is a meta tool that combines five methods (SIFT, PhD-SNP, PolyPhen-2, SNAP and I-Mutant 3.0) to predict the probability that a nsSNP will affect protein function and may consequently be disease-related. It utilizes a machine learning-based method (RF) for predicting whether variants affect functions and thereby lead to diseases. The PON-P classifies the nsSNPs as neutral, unclassified or pathogenic with a corresponding probability of pathogenicity, and provides the data available in the Uniprot database for each entry [56].

PredictSNP1.0 is a SNP classifier tool that combines six prediction methods (MAPP, PhD-SNP, PolyPhen-1, PolyPhen-2, SIFT and SNAP) to obtain a consensus prediction of the effect of the amino acid substitution. The six prediction tools are run using a dataset of non-redundant mutations. The individual confidence scores are transformed to percentages to allow comparison, and the individual predictions are combined in the consensus prediction. The predictions are supplemented by experimental annotations from Protein Mutant Database and Uniprot [31].

In order to identify the nsSNPs more probably damaging in the gene the categorical prediction of the individual tools were combined by the count of damage results and the nsSNPs were classified from the most neutral (no damaging results) to the most damaging (damaging prediction in the eleven tools).

### Statistical analysis

The Pearson correlation coefficients between the prediction scores for deleterious effect or the probability of pathogenicity provided by the programs SIFT, Polyphen-2, PROVEAN, MutPred, PANTHER, SNPs3D and Mutation Assessor were analyzed. The associations among the neutral or damaging results of the categorical classification of the prediction tools were evaluated by Chi-square test (χ^2^) for independence by contingency table analysis. The statistical significance of differences in the combine of damaging results of individual tools in the domains of the *MC1R* protein were evaluated by the Kruskal-Wallis test. The statistical analyses were performed in the SPSS v. 20 program (IBM Corp., Armonk, NY, USA).

## Results

### Prediction Programs

A total of 92 nsSNPs from the NCBI dbSNP database were analyzed to identify the deleterious mutations. Of these, 76 were found to be damaging (score < 0.05) by SIFT, with 38 assigned a score of 0.

The PROVEAN score was lower than-2.5 for 51 nsSNPs, indicating that these variants do affect the protein function and are likely to be deleterious.

In Polyphen-2, a total of 54 nsSNPs were predicted as damaging (PSIC > 0.5); 12 of these nsSNPs were predicted to be highly deleterious, with a PSIC score of 1.

In the MutPred analysis, 57 nsSNPs showed a probability of being a deleterious mutation, with *g* scores higher than 0.5. For 22 of these nsSNPs the program indicated an actionable or confident hypothesis (*p* score < 0.05) that the molecular mechanism would be disrupted.

The PANTHER software estimates the likelihood that the nsSNPs will affect the function of the protein [50]. The calculated subPSECs were equal to or lower than-3, resulting in a probability of deleterious effect higher than 0.5 for 43 nsSNPs.

The DDG predicted by I-Mutant 3.0 classified 86 of the nsSNPs as decreasing the stability of the mutated protein (DDG <0) and 6 as increasing it (DDG>0). We used the sequence-based tool of the I-Mutant 3.0 suite to predict the disease-associated nsSNPs. A total of 73 nsSNPs were predictted to be disease-related by this method.

According to the Mutation Assessor analysis, 15 nsSNPs showed a high functional impact score (FI), 48 a medium score, and 21 had a low functional impact; 8 were neutral (High: FI > 3.5 / Low: 0.8 < FI ≤ 1.9 / Medium: 1.9 < FI ≤ 3.5 / Neutral: FI ≤ 0.8).

A negative SVM score in SNPs3D was obtained for 49 nsSNPs, indicating a variant classified as deleterious; the other 43 nsSNPs received a positive score, which indicates a likely non-deleterious mutation.

**Fig 1:**
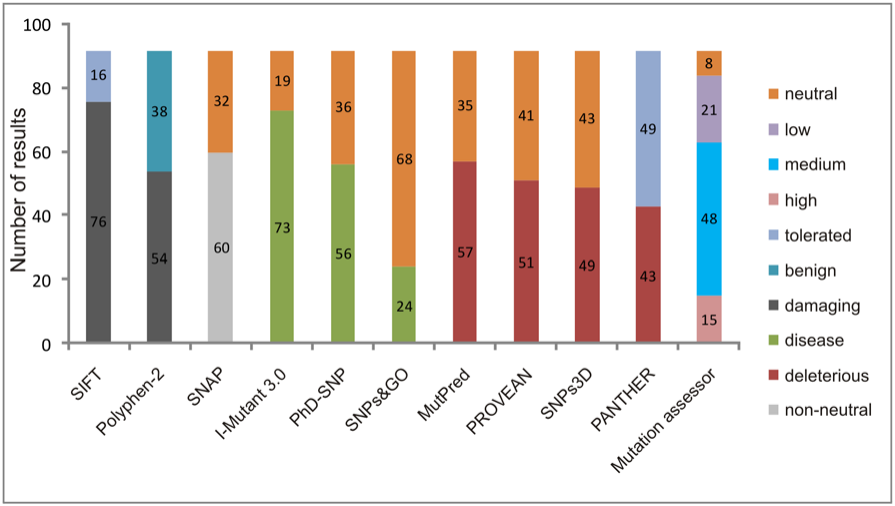
Prediction results of the 92 nsSNPs in the *MC1R* gene analyzed by the 11 tools. The different categorical classifications of the 11 tools are showed.

The PhD-SNP 2.0 and SNPs&GO tools classify the mutation as a disease-related or neutral polymorphism. Of the set of nsSNPs in the *MC1R* gene analyzed, 56 were predicted to be disease-related by PhD-SNP 2.0, and the SNPs&GO method classified 24 nsSNPs as disease-related. The SNAP method indicated that 60 nsSNPs were functionally non-neutral. The prediction results of the 11 tools are summarized in Fig. 1.

The deleterious scores from SIFT, Polyphen-2, PROVEAN, MutPred, PANTHER, SNPs3D and Mutation Assessor, provide a numerical value associated with the prediction. In Polyphen-2, MutPred, and Mutation Assessor highers scores indicate damaging mutations, while in SIFT, PROVEAN, PANTHER, SNPs3D lower or negative scores correspond to damaging SNPs. These differences in the score results in negative values of the correlation coeficient between tools with inverse mathematical signal. Considering the absolute value of the Pearson coefficients the tools showed significant correlation with each other with R^2^ ranging from 0.276 between SIFT and MutPred to 0.755 between SNPs3D and Mutation Assessor (Table 2).

**Table 2:** Matrix of Pearson correlation between the prediction tools.

	Polyphen-2	PROVEAN	MutPred	PANTHER	SNPs3D	Mutation Assessor
SIFT	-0.390*	0.441*	-0.276*	0.361*	0.508*	-0.578*
POLYPHEN-2	-	-0.629*	0.323*	-0.583*	-0.700*	0.619*
PROVEAN		-	-0.351*	0.740*	0.705*	-0.711*
MutPred			-	-0.294*	-0.390*	0.368*
PANTHER				-	0.610*	-0.662*
SNPs3D					-	-0.755*
Mutation assessor						-

* significative association with p<0.05.

The majority of the 11 tools had a significant association between their categorical prediction results (Chi-square test for independence—P<0.05), with the exception of I-Mutant 3.0, which showed a significant association only with SNPs&GO (Table 3).

**Table 3:** Matrix of Chi-square analysis of association between the prediction tools results.

Tool	Polyphen-2	PROVEAN	MutPred	PANTHER	I-Mutant 3.0	SNPs3D	PhD-SNP	SNP&GO	Mutation Assessor	SNAP
SIFT	21.974	10.551	11.223	9.121	**0.223**	12.927	14.426	6.836	5.487	13.810
Polyphen-2	-	26.418	5.845	24.912	**0.197**	31.568	15.692	18.472	20.861	18.914
PROVEAN		-	7.651	22.025	**3.351**	33.840	31.010	21.451	16.791	31.477
MUTPRED			-	10.031	**0.168**	13.833	10.330	**2.344**	17.180	9.472
PANTHER				-	**0.942**	21.603	21.486	31.438	22.533	15.441
I-Mutant 3.0					-	**1.197**	**3.540**	5.385	**0.001**	**1.672**
SNPs3D						-	31.815	19.240	22.071	32.748
PhD-SNP							-	16.665	12.380	22.088
SNP&GO								-	11.256	13.417
Mutation Assessor									-	17.635

The results in bold were not significant (P>0.05).

**Fig 2:**
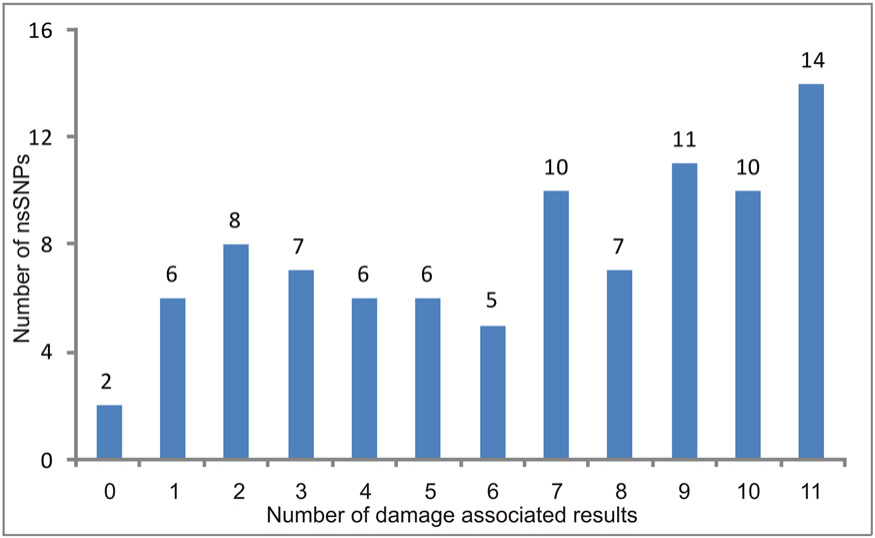
Distribution of the count of damage results of the 11 tools in the nsSNPs in *MC1R* gene.

The results of the 11 prediction tools were combined in order to identify the most damage nsSNPs in the *MC1R* gene. A total of 57 nsSNPs (about 62%) were predicted as damaging by more than five tools (Fig. 2).

**Fig 3:**
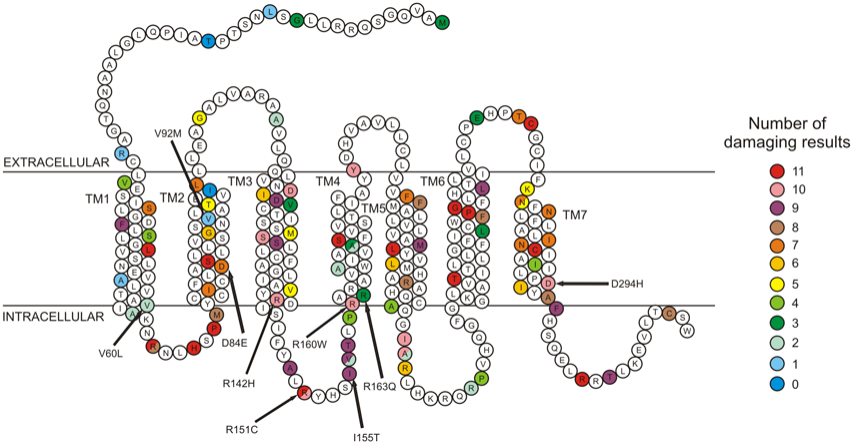
Two-dimensional structure of the *MC1R* protein according to the reference sequence of the *MC1R* gene (NP_002377). One letter amino acid code is used. The 92 nsSNPs analyzed are colored in relation to the count of damage results in the 11 tools (legend). The RHC associated mutations are indicated by the arrows. TM: transmembrane domains.

The numbers of damage results in the 11 tools for the 92 nsSNPs in the *MC1R* protein are represented in Fig. 3. Two nsSNPs (T19I and I98V) showed neutral results in all tools. A total of 14 nsSNPs (L48P, R67W, H70Y, P72L, S83P, R151H, S172I, L206P, T242I, G255R, P256S, C273Y, C289R and R306H) present damage results in all the prediction methods, likely a harmful variation in the gene.

The prediction scores of the tools indicate differences between the nsSNPs selected as damaging by the 11 tools. Among the 14 nsSNPs, 12 showed a SIFT score of 0, and six (L48P, R67W, R151H, L206P, P256S and C273Y) showed a Polyphen-2 PSIC score of 1, indicating that they may be highly damaging mutations. The MutPred tool indicated hypotheses of the molecular mechanisms disrupted (*g* score >0.5 and *p* score <0.05) by the nsSNPs L48P, R67W, R151H, S172I, L206P and C273Y, including loss of solvent accessibility, loss of catalytic residue, loss of stability, and gain of methylation (Table 4). The nsSNP C273Y showed the highest deleterious scores of the mutations in the SIFT, Polyphen-2, PANTHER, PROVEAN and MutPred programs, demonstrating the concordance of the results from the different tools used to predict the most damaging polymorphisms in the *MC1R* gene.

**Table 4:** Prediction scores from SIFT, PROVEAN, Polyphen-2, PANTHER, SNPs3D, Mutation Assessor and MutPred tools of the nsSNPs selected as the most damaging in *MC1R* gene.

SNP ID	Mutation	SIFT score	PROVEANsc ore	PolyPhen-2 PSIC score	PANTHER		SNPs3D SMV score	Mutation assessor		MutPred	
					subPSEC	Pdeleterious		FIS score	Functional Impactor	*g* score	Molecular Mechanism Disrupted (P)
rs201787533	L48P	0	-6.202	1	-4.77696	0.85532	-2.64	3.250	medium	0.717	Loss of catalytic residue at L48 (P = 0.0274)
rs372590533	R67W	0	-5.538	1	-5.53364	0.92647	-1.19	3.880	high	0.535	Loss of solvent accessibility (P = 0.0087)
rs377122753	H70Y	0	-5.526	0.996	-3.71378	0.67123	-1.61	3.785	high	0.746	
rs377297107	P72L	0	-9.182	0.997	-6.50925	0.97095	-1.07	3.055	medium	0.767	
rs34474212	S83P	0.001	-3.287	0.999	-3.35336	0.58743	-1.06	3.800	high	0.759	
rs149922657	R151H	0	-4.533	1	-4.31313	0.78804	-0.59	2.580	medium	0.542	Loss of solvent accessibility (P = 0.0299)
rs376670171	S172I	0	-3.222	0.996	-4.19542	0.76771	-0.71	4.190	high	0.759	Loss of glycosylation at S172 (P = 0.0252)
rs377499038	L206P	0	-6.564	1	-6.45286	0.96932	-2.64	3.910	high	0.824	Loss of stability (P = 0.0428) *
rs200051702	T242I	0	-5.619	0.999	-3.53343	0.63028	-1.61	3.620	high	0.815	
rs371214731	G255R	0	-4.036	0.992	-3.60908	0.64773	-0.78	3.175	medium	0.824	
rs200215218	P256S	0	-7.311	1	-5.89441	0.94757	-1.96	4.255	high	0.844	
rs368281517	C273Y	0	-9.733	1	-6.92057	0.98056	-0.59	3.530	high	0.854	Gain of methylation at K278 (P = 0.0482) *
rs369542041	C289R	0	-8.928	0.981	-3.52876	0.62919	-1.37	3.380	medium	0.885	
rs368507952	R306H	0.001	-3.680	0.999	-5.97409	0.95139	-1.75	3.835	high	0.799	

(*)The molecular mechanism disrupted show the actionable hypothesis when the probability of deleterious mutation (g score) are bigger than 0.5 and the probability of impacted structural or functional properties (p score) are < 0.05.

The distribution of the prediction results was not equal along the protein: 18 nsSNPs occur in the extracellular domain, 28 in the intracellular domain, and 46 in the transmembrane domain. The number of damaging results was significantly lower in the extracellular domain (mean = 4.22±3.26) in relation to the transmembrane (mean = 6.89±3.17) and intracellular (mean = 7.6±3.28) domains (Kruskal-Wallis Test H: 10.978, P = 0.004, df = 2). The different transmembrane domains did not show significant differences in the number of damaging results of the nsSNPs (Kruskal-Wallis Test H: 6.84, P = 0.336, df = 6).

### Analysis of consensus prediction tools

The PredictSNP 1.0 and PON-P consensus tools predicted 58 and 20 nsSNPs as deleterious and pathogenic, respectively (S1 Table). The PON-P gave unclassified results for 36 nsSNPs. The two consensus analysis tools showed a significant association among these (χ^2^: 36.823, p<0.05).

While most of the nsSNPs with more than five damaging results coincided with PredictSNP 1.0 classifications, three nsSNPs that were classified as deleterious (S41C, I120T and I297V) were predicted as neutral in PredictSNP 1.0, and four (M1I, M128T, K278E, and I292T) with less than five damaging results were classified as deleterious in the PredictSNP 1.0 analysis.

Of the 57 nsSNPs classified as deleterious by more than five tools, 20 were predicted as pathogenic, 30 as unclassified and 7 as neutral by PON-P; while of the 35 nsSNPs classified as neutral in the combine analysis, 29 were also classified as neutral in PON-P and six were predicted as unclassified.

## Discussion

### Determination of the most damaging nsSNPs

The non-synonymous polymorphisms situated in the *MC1R* gene were evaluated by 11 programs that use different methods to predict the damaging nsSNPs. The differences in the predictions generated by the programs indicate the need for a combined analysis that could identify with accuracy the nsSNPs that are most damaging to the function of the *MC1R* gene.

For this purpose we combined the results of the 11 tools to classify the nsSNPs from, the most neutral to the more damaging. The majority of the nsSNPs (57, about 62%) were predicted as damaging, deleterious or disease-associated by more than five programs showing high concordance with two consensus prediction tools (Fig. 2).

The 14 nsSNPs classified as deleterious in the 11 tools were selected as the most damaging in our combined analysis and were predicted as deleterious by PredictSNP 1.0, and as pathogenic or unclassified by PON-P (S1 Table). Among the 14 nsSNPs only C289R (rs369542041) has been previously analyzed in the literature [8] showing absence of functional coupling to the cAMP pathway, and being unable to bind to agonist efficiently. The C273Y nsSNP that presents higher scores in five of the 11 tools are localized in the third extracellular loop domain (Fig. 3) and affects a cystein highly conserved in *MC1R* gene across different species, according to MSA analysis in Polyphen-2, PANTHER and Mutation Assessor. Although the majority of the 14 nsSNPs most damaging described here were not analyzed by *in vitro* tests and there is no information on the functional significance of these mutations in *MC1R* protein the results demonstrated that these can be prioritized in further populational and laboratory studies.

The strategy of use the predictions of different tools was utilized to analyze the nsSNPs in different genes involved in biological processes, allowing the most deleterious mutations to be selected. The combination of tools resulted in the indication of four, two and one nsSNPs as the most deleterious mutations in the TYR, TYRP1 and P proteins of the gene, which are associated with oculocutaneous albinism type IA (*OCA1A*) [41], type III (*OCA3*) [42] and type II (*OCA2*) [40], respectively. These results demonstrate that the use of a combination of tools could adjust for the differences between the programs and improve the accuracy of the search for the important polymorphisms, the occurrence of diseases or the phenotype variations.

### Analysis of Red Hair Color (RHC) and Pathogenic MC1R variants

The *MC1R* gene has been associated with variation in human skin and hair pigmentation, UV-induced skin damage, and cutaneous malignant melanoma. The red hair color (RHC) phenotype is due to the production of more pheomelanin than eumelanin, and is usually a result of *MC1R* recessive alleles that impair the function of the receptor [57] [58]. The variants D84E, R151C, R160W and D294H are strongly associated with red hair and fair skin phenotypes, and are classified as high-penetrance R alleles; while the variants V60L, V92M, and R163Q have low penetrance in these features and are classified as r alleles [6] [8] [59] [60] [61] [62]. The variants R142H and I155T are less frequent and have also been associated with RHC, based on findings of a strong family association. R142H shows an association with RHC that is similar to the other R alleles, while the association of I155T was low in a meta-analysis [63].

Additionally, some polymorphisms (V60L, D84E, V92M, R142H, R151C, I155T, R160W, R163Q and D294H) were identified as involved in elevated risk of the development of melanoma [63] [64] [65] [66] [67] [68]. The available information in the NCBI and Uniprot databases about nsSNPs that are classified as pathogenic is listed in S2 Table.

The polymorphisms characterized as RHC-associated or pathogenic in the dbSNP database R142H, R151C, R160W and D294H were predicted as having damaging effects in 10 of the 11 programs, I155T in nine programs and D84E in seven programs (Fig. 3 and S2 Table). These six polymorphisms were classified as deleterious in the two consensus analyses (S1 Table).

The nsSNP R163Q was predicted as damaging in three programs, and V60L in two. The V92M mutation was classified as damaging only in I-Mutant 3.0. Those three nsSNPs were predicted as neutral in PredictSNP and PON-P consensus analyses.

Kanetsky et al. [69] found a concordance between the RHC categories of the *MC1R* variants and the prediction of damaging changes, by means of an evolutionary amino acid conservation approach using SIFT. The R alleles D84E, R142H, R151C, I155T, R160W and D284H were predicted to be intolerant, and the variants V60L, V92M and R163Q were predicted to be tolerant. Their categories defined by SIFT gave similar results in the analysis of association with phenotypes in relation to the literature classification in a Caucasian population. Zhang et al. [70] analyzed a set of 22 nsSNPs in *MC1R* with SIFT and Polyphen, and found that the two programs classified 11 as damaging, including the R variants.

The variation in the prediction results of nsSNPs indicated in the literature classification as major (R) and minor (r) associated with the RHC phenotype [71] [72] [73], [74], [75] highlight the need for laboratory studies of the functional effects of the other nsSNPs predicted as damaging in the *MC1R* gene.

## Conclusion

The analysis of the SNP involved in the determination of variation in phenotypes or in complex diseases is a challenge that requires different approaches. Here, we used different methods to predict the most damaging mutations in the human *MC1R* gene, a key protein in the control of pigmentation in animals. Although some of the polymorphisms found in *MC1R* have been studied in the laboratory, many others have not yet been evaluated with respect to their possible damaging effects on protein structure and function.

The programs used here are based on evolutionary, structural and computational methods, gathering information on these different properties of the alterations caused by the mutations and predicting those that are most probably damaging or disease-associated. The analysis of the results demonstrated the association between the different methods employed, with the consensus tools supporting the strategies applied to the discrimination of the damaging from the neutral nsSNPs.

Our characterization of the nsSNPs as damaging or neutral based in the combination of the tools indicate differences in the damaging prediction of the RHC-associated alleles classified in the literature as high-penetrance (R) or low-penetrance (r) alleles, although it was not clear what mechanism or mechanisms are involved in the differences in the effects of these alleles. The selected most-probably damaging nsSNPs could be prioritized in further studies of the functional properties of the mutated receptor. In particular, the C273Y polymorphism, located in the third extracellular loop, was indicated as the most deleterious by different tools.

Finally, these results may contribute to the understanding of the variations in skin and hair phenotypes, and of the causes of complex diseases such as melanoma.

## Supporting Information

S1 Table Prediction results of the nsSNPs in *MC1R* human gene. Results of the eleven individual tools, of the two consensus tools PON-P and PredictSNP 1.0. The nsSNPs in bold were selected by filter analysis.
(DOC)

S2 Table Information available about the *MC1R* nsSNPs. The data in dbSNP (NCBI) and Uniprot databases about the nsSNPs classified as pathogenic and the alleles associated with RHC phenotype in literature. R: alleles with high penetrance; r: alleles with low penetrance in RHC. * alleles with divergences in the RHC classification.
(DOC)

## References

[1] Cone RD, Lu D, Koppula S, Väge DI, Klungland H, Boston B, et al The melanocortin receptors: agonists, antagonists, and the hormonal control of pigmentation. Recent Prog Horm Res. 1996;51: 287–317. 8701084

[2] Beaumont KA, Wong SS, Ainger SA, Liu YY, Patel MP, Millhauser GL, et al Melanocortin MC1 receptor in human genetics and model systems. Eur J Pharmacol. 2011;660: 103–110. 10.1016/j.ejphar.2010.11.040 21199646 PMC3095693

[3] Valverde P, Healy E, Jackson I, Rees JL, Thody AJ. Variants of the melanocyte-stimulating hormone receptor gene are associated with red hair and fair skin in humans. Nat Genet. 1995;11: 328–330. 7581459 10.1038/ng1195-328

[4] Schioth HB, Phillips SR, Rudzish R, Birch-Machin MA, Wikberg JE, Rees JL. Loss of function mutations of the human melanocortin 1 receptor are common and are associated with red hair. Biochem Biophys Res Commun 1999;260: 488–491. 10403794 10.1006/bbrc.1999.0935

[5] Beaumont KA, Newton RA, Smit DJ, Leonard JH, Stow JL, Sturm RA. Altered cell surface expression of human *MC1R* variant receptor alleles associated with red hair and skin cancer risk. Hum Mol Genet. 2005;14: 2145–2154. 15972726 10.1093/hmg/ddi219

[6] Beaumont KA, Shekar SN, Newton RA, James MR, Stow JL, Duffy DL, et al Receptor function, dominant negative activity and phenotype correlations for *MC1R* variant alleles. Hum Mol Genet. 2007;16: 2249–2260. 17616515 10.1093/hmg/ddm177

[7] Fernandez LP, Milne RL, Bravo J, Lopez JM, Avilés JA, Longo MI, et al *MC1R*: three novel variants identified in a malignant melanoma association study in the Spanish population. Carcinogenesis. 2007;28: 1659–1664. 17434924 10.1093/carcin/bgm084

[8] Pérez-Oliva AB, Fernéndez LP, Detorre C, Herráiz C, Martínez-Escribano JA, Benítez J, et al Identification and Functional Analysis of Novel Variants of the Human Melanocortin 1 Receptor Found in Melanoma Patients. Hum Mutat. 2009;30: 811–822. 10.1002/humu.20971 19338054

[9] Scherer D, Nagore E, Bermejo JL, Figl A, Botella-Estrada R, Thirumaran RK, et al Melanocortin receptor 1 variants and melanoma risk: A study of 2 European populations. Int J Cancer. 2009;125: 1868–1875. 10.1002/ijc.24548 19585506

[10] Thusberg J, Vihinen M. Pathogenic or Not? And if So, Then How? Studying the Effects of Missense Mutations Using Bioinformatics Methods. Hum Mutat. 2009;30: 703–709. 10.1002/humu.20938 19267389

[11] Vendruscolo M, Zurdo J, MacPhee CE, Dobson CM. Protein folding and misfolding: a paradigm of self-assembly and regulation in complex biological systems. Phil. Trans. R. Soc. Lond. A. 2003;361: 1205–1222. 10.1098/rsta.2003.1194 12816607

[12] Hichiya H, Tanaka-Kagawa T, Soyama A, Jinno H, Koyano S, Katori N, et al Functional Characterization of Five Novel CYP2C8 Variants, G171S, R186X, R186G, K247R and K383N, Found in a Japanese Population. Drug Metab Dispos. 2005;33: 630–636. 15716363 10.1124/dmd.105.003830

[13] Josephy PD, Kent M, Mannervik B. Single-nucleotide polymorphic variants of human glutathione transferase T1–1 differ in stability and functional properties. Arch Biochem Biophys. 2009;490: 24–29. 10.1016/j.abb.2009.07.025 19664997

[14] Gorlatova N, Chao K, Pal LR, Galkin A, Araj RH, Turko I, et al Protein Characterization of a Candidate Mechanism SNP for Crohn’s Disease: The Macrophage Stimulating Protein R689C Substitution. PLoS ONE. 2011;6: e27269 10.1371/journal.pone.0027269 22087277 PMC3210151

[15] Esaki S, Malkaram SA, Zempleni J. Effects of single-nucleotide polymorphisms in the human holocarboxylase synthetase gene on enzyme catalysis. Eur J Med Genet 2012;20: 428–433. 10.1038/ejhg.2011.198 PMC3306849 22027809

[16] Zeron-Medina J, Wang X, Repapi E, Campbell MR, Su D, Castro-Giner J, et al A Polymorphic p53 Response Element in KIT Ligand Influences Cancer Risk and Has Undergone Natural Selection. Cell. 2013;155: 410–422. 10.1016/j.cell.2013.09.017 24120139 PMC4171736

[17] Morisseau C, Wecksler AT, Deng C, Dong H, Yang J. Effect of soluble epoxide hydrolase polymorphism on substrate and inhibitor selectivity and dimer formation. J Lipid Res. 2014;55: 1131–1138. 24771868 10.1194/jlr.M049718 PMC4031944

[18] Valastyan JS, Lindquist S. Mechanisms of protein-folding diseases at a glance. Dis Model Mech. 2014;7: 9–14. 10.1242/dmm.013474 24396149 PMC3882043

[19] Mooney S. Bioinformatics approaches and resources for single nucleotide polymorphism functional analysis. Brief Bioinform. 2005;6: 44–56. 15826356 10.1093/bib/6.1.44

[20] Bishop DT, Demenais F, Iles MM, Harland M, Taylor JC, Corda E, et al Genome-wide association study identifies three loci associated with melanoma risk. Nat Genet. 2009;41: 920–928. 10.1038/ng.411 19578364 PMC2741419

[21] Barrett JH, Iles MM, Harland M, Taylor JC, Aitken JF, Andresen PA, et al Genome-wide association study identifies three new melanoma susceptibility loci. Nat Genet. 2011;43: 1108–1114. 10.1038/ng.959 21983787 PMC3251256

[22] Liu CT, Garnaas MK, Tin A, Kottgen A, Franceschini N, Peralta CA, et al Genetic Association for Renal Traits among Participants of African Ancestry Reveals New Loci for Renal Function. PLoS Genet. 2011;7: e1002264 10.1371/journal.pgen.1002264 21931561 PMC3169523

[23] Bush WS, Moore JH. Chapter 11: Genome-Wide Association Studies. PloS Comput Biol 2012;8: e1002822 10.1371/journal.pcbi.1002822 23300413 PMC3531285

[24] Gorski M, Tin A, Garnaas M, McMahon GM, Chu AY, Tayo BO, et al. Genome-wide association study of kidney function decline in individuals of European descent. Kidney Int. 2014; 10.1038/ki.2014.361 PMC4425568 25493955

[25] Ohashi J, Tokunaga K. The power of genome-wide association studies of complex disease genes: statistical limitations of indirect approaches using SNP markers. J Hum Genet. 2001;46: 478–482. 11501946 10.1007/s100380170048

[26] Klein RJ. Power analysis for genome-wide association studies. BMC Genet. 2007;8:10.1186/1471-2156-8-58 PMC2042984 17725844

[27] Spencer CCA, Su Z, Donnelly P, Marchini J. Designing Genome-Wide Association Studies: Sample Size, Power, Imputation, and the Choice of Genotyping Chip. PLoS Genet. 2009;5: e10000477. 10.1371/journal.pgen.1000477 PMC2688469 19492015

[28] Kumar A, Rajendran V, Sethumadhavan R, Shukla P, Tiwari S, Purohit R. Computational SNP Analysis: Current Approaches and Future Prospects. Cell Biochem Biophys. 2014;68: 233–239. 10.1007/s12013-013-9705-6 23852834

[29] Ng PC, Henikoff S. Predicting the Effects of Amino Acid Substitutions on Protein Function. Annu Rev Genomics Hum Genet. 2006;7: 61–80. 16824020 10.1146/annurev.genom.7.080505.115630

[30] Yue P, Moult J. Identification and Analysis of Deleterious Human SNPs. J Mol Biol. 2006;356: 1263–1274. 16412461 10.1016/j.jmb.2005.12.025

[31] Bendl J, Stourac J, Salanda O, Pavelka A, Wieben ED, Zendulka J, et al PredictSNP: Robust and Accurate Consensus Classifier for Prediction of Disease-Related Mutations. PLoS Comput Biol. 2014;10: e1003440 10.1371/journal.pcbi.1003440 24453961 PMC3894168

[32] Rajasekaran R, Sudandiradoss C, Doss CGP, Sethumadhavan R. Identification and *in silico* analysis of functional SNPs of the BRCA1 gene. Genomics. 2007;90: 447–452. 17719744 10.1016/j.ygeno.2007.07.004

[33] Kumar A, Rajendran V, Sethumadhavan R, Purohit R. In silico prediction of a disease-associated STIL mutant and its affect on the recruitment of centromere protein J (CENPJ). FEBS Open Bio. 2012;2: 285–293. 10.1016/j.fob.2012.09.003 23772360 PMC3678130

[34] Kumar A, Purohit R. Computational screening and molecular dynamics simulation of disease associated nsSNPs in CENP-E. Mutat Res. 2012;738–739: 28–37. 10.1016/j.mrfmmm.2012.08.008 22974711

[35] Doss CGP, Sudandiradoss C, Rajasekaran R, Purohit R, Ramanathan K, Sethumadhavan R, et al Identification and structural comparison of deleterious mutations in nsSNPs of ABL1 gene in chronic myeloid leukemia: A bio-informatics study. J Biomed Inform. 2008;41: 607–612. 10.1016/j.jbi.2007.12.004 18243808

[36] Brunham LR, Singaraja RR, Pape TD, Kejariwal A, Thomas PD, Hayden MR. Accurate Prediction of the Functional Significance of Single Nucleotide Polymorphisms and Mutations in the ABCA1 Gene. PLoS Genet. 2005;1: e83 16429166 10.1371/journal.pgen.0010083 PMC1342637

[37] Carvalho MDC, Mesquita JF. Structural Modeling and In Silico Analysis of Human Superoxide Dismutase 2. PLoS One. 2013;8: e65558 10.1371/journal.pone.0065558 23785434 PMC3681941

[38] Moreira LGA, Pereira LC, Drummond PR, Mesquita JF. Structural and Functional Analysis of Human SOD1 in Amyotrophic Lateral Sclerosis. PLoS One. 2013;8: e81979 10.1371/journal.pone.0081979 24312616 PMC3846731

[39] Vanajothi R, Rajamanikandan S, Sudha A, Srinivasan P. Structural and functional analysis of KIT gene encoding receptor tyrosinase and its interaction with sunitinib and HDAC inhibitors: an in silico approach. Pak J Biol Sci. 2012;15: 121–131. 22866542 10.3923/pjbs.2012.121.131

[40] Kamaraj B, Purohit R. Computational Screening of Disease-Associated Mutations in OCA2 Gene. Cell Biochem Biophys. 2014;68: 97–109. 10.1007/s12013-013-9697-2 23824587

[41] Kamaraj B, Purohit R. Mutational analysis of TYR gene and its structural consequences in OCA1A. Gene. 2013;513: 184–195. 10.1016/j.gene.2012.09.128 23085273

[42] Kamaraj B, Purohit R. *In silico* screening and molecular dynamics simulation of disease-associated nsSNP in TYRP1 gene and its structural consequences in OCA3. Biomed Res Int. 2013;697051 10.1155/2013/697051 23862152 PMC3703794

[43] Ng PC, Henikoff S. SIFT: predicting amino acid changes that affect protein function. Nucleic Acids Res. 2001;31: 3812–3814. 10.1093/nar/gkg509 PMC168916 12824425

[44] Li B, Krishnan VG, Mort ME, Xin F, Kamati KK, Cooper DN, et al Automated inference of molecular mechanisms of disease from amino acid substitutions. Bioinformatics. 2009;25: 2744–2750. 10.1093/bioinformatics/btp528 19734154 PMC3140805

[45] Adzhubei IA, Schmidt S, Peshkin L, Ramensky VE, Gerasimova A, Bork P, et al A method and server for predicting damaging missense mutations. Nat Methods. 2010;7: 248–249. 10.1038/nmeth0410-248 20354512 PMC2855889

[46] Choi Y, Sims GE, Murphy S, Miller JR, Chan AP. Predicting the Functional Effect of Amino Acid Substitutions and Indels. PLoS ONE. 2012;7: e46688 10.1371/journal.pone.0046688 23056405 PMC3466303

[47] Choi Y. A Fast Computation of Pairwise Sequence Alignment Scores Between a Protein and a Set of Single-Locus Variants of Another Protein In Proceedings of the ACM Conference on Bioinformatics, Computational Biology and Biomedicine (BCB ′12). ACM, New York, NY 2012 pp. 414–417.

[48] Capriotti E, Fariselli P, Rossi I, Casadio R. A three-state prediction of single point mutations on protein stability changes. BMC Bioinformatics. 2008;9: S6 10.1186/1471-2105-9-S12-S6 18387208 PMC2323669

[49] Thomas PD, Campbell MJ, Kejariwal A. PANTHER: A Library of Protein Families and Subfamilies Indexed by Function. Genome Res. 2003;13: 2129–2141. 12952881 10.1101/gr.772403 PMC403709

[50] Thomas PD, Kejariwal A. Coding single-nucleotide polymorphisms associated with complex vs. Mendelian disease: Evolutionary evidence for differences in molecular effects. PNAS. 2004;101: 15398–15403. 15492219 10.1073/pnas.0404380101 PMC523449

[51] Yue P, Melamud E, Moult J. SNPs3D: Candidate gene and SNP selection for association studies. BMC Bioinformatics. 2006;7: 166 16551372 10.1186/1471-2105-7-166 PMC1435944

[52] Reva B, Antipin Y, Sander C. Predicting the functional impact of protein mutations: application to cancer genomics. Nucleic Acids Res. 2011;39: e118 10.1093/nar/gkr407 21727090 PMC3177186

[53] Capriotti E, Calabrese R, Casadio R. Predicting the insurgence of human genetic diseases associated to single point protein mutations with support vector machines and evolutionary information. Bioinformatics. 2006;22: 2729–2734. 16895930 10.1093/bioinformatics/btl423

[54] Calabrese R, Capriotti E, Fariselli P, Martelli PL, Casadio R. Functional Annotations Improve the Predictive Score of Human Disease-Related Mutations in Proteins. Hum Mutat. 2009;30: 1237–1244. 10.1002/humu.21047 19514061

[55] Bromberg Y, Rost B. SNAP: predict effect of non-synonymous polymorphisms on function. Nucleic Acids Res. 2007;35: 3823–3835. 17526529 10.1093/nar/gkm238 PMC1920242

[56] Olatubosun A, Väliaho J, Härkönen J, Thusberg J, Vihinen M. PON-P: Integrated Predictor for Pathogenicity of Missense Variants. Hum Mutat. 2012;33: 1166–1174. 10.1002/humu.22102 22505138

[57] Rees JL. Genetics of Hair and Skin Color. Annu Rev Genet. 2003;37: 67–90. 14616056 10.1146/annurev.genet.37.110801.143233

[58] Wong TH, Rees JL. The relation between melanocortin 1 receptor (*MC1R*) variation and the generation of phenotypic diversity in the cutaneous response to ultraviolet radiation. Peptides. 2005;26: 1965–1971. 15963603 10.1016/j.peptides.2004.11.021

[59] Flanagan N, Healy E, Ray A, Philips S, Todd C, Jackson IJ, et al Pleiotropic effects of the melanocortin 1 receptor (*MC1R*) gene on human pigmentation. Hum Mol Genet. 2000;9: 2531–2537. 11030758 10.1093/hmg/9.17.2531

[60] Duffy DL, Box NF, Chen W, Palmer JS, Montgomery GW, James MR, et al Interactive effects of *MC1R* and OCA2 on melanoma risk phenotypes. Hum Mol Genet. 2004;13: 447–461. 14709592 10.1093/hmg/ddh043

[61] Sturm RA, Duffy DL, Box NF, Newton RA, Shepherd AG, Chen W, et al Genetic Association and Cellular Function of *MC1R* Variant Alleles in Human Pigmentation. Ann N Y Acad Sci. 2003;994: 348–358. 12851335 10.1111/j.1749-6632.2003.tb03199.x

[62] Sulem P, Gudbjartsson DF, Stacey SN, Helgason A, Rafnar T, Magnusson KP, et al Genetic determinants of hair, eye and skin pigmentation in Europeans. Nat Genet. 2007;39: 1443–1452. 17952075 10.1038/ng.2007.13

[63] Raimondi S, Sera F, Gandini S, Iodice S, Caini S, Maisonneuve P, et al *MC1R* variants, melanoma and red hair color phenotype: a meta-analysis. Int J Cancer. 2008;122: 2753–2760. 10.1002/ijc.23396 18366057

[64] Bastiaens MT, Huurne JAC, Kielich C, Gruis NA, Westendorp RGJ, Vermeer RGJ, et al Melanocortin-1 Receptor Gene Variants Determine the Risk of Nonmelanoma Skin Cancer Independently of Fair Skin and Red Hair. Am J Hum Genet. 2001;68: 884–894. 11254446 10.1086/319500 PMC1275642

[65] Kennedy C, Huurne J, Berkhout M, Gruis N, Bastiaens M, Bergman W, et al Melanocortin 1 Receptor (*MC1R*) Gene Variants are Associated with an Increased Risk for Cutaneous Melanoma Which is Largely Independent of Skin Type and Hair Color. J Invest Dermatol. 2001;117: 294–300. 11511307 10.1046/j.0022-202x.2001.01421.x

[66] Scott MC, Wakamatsu K, Ito S, Kadekaro AL, Kobayashi N, Groden J, et al Human melanocortin 1 receptor variants, receptor function and melanocyte response to UV radiation. J Cell Sci. 2002;115: 2349–2355. 12006619 10.1242/jcs.115.11.2349

[67] Koppula SV, Robbins LS, Lu D, Baack E, White CR Jr, Swanson NA, et al Identification of Common Polymorphisms in the Coding Sequence of the Human MSH Receptor (*MC1R*) With Possible Biological Effects. Hum Mutat. 1997;9: 30–36. 8990005 10.1002/(SICI)1098-1004(1997)9:1<30::AID-HUMU5>3.0.CO;2-T

[68] Ringholm A, Klovins J, Rudzish R, Phillips S, Rees JL, Schiöth HB. Pharmacological Characterization of Loss of Function Mutations of the Human Melanocortin 1 Receptor That Are Associated with Red Hair. J Investig Dermatol. 2004;123: 917–923. 15482480 10.1111/j.0022-202X.2004.23444.x

[69] Kanetsky PA, Ge F, Najarian D, Swoyer J, Panossian S, Schuchter L, et al Assessment of Polymorphic Variants in the Melanocortin-1 Receptor Gene with Cutaneous Pigmentation Using an Evolutionary Approach. Cancer Epidemiol Biomarkers Prev. 2004;13: 808–819. 15159314

[70] Zhang CS, Geng LY, Liu ZZ, Fu ZX, Gong YF, Feng MS, et al A comprehensive in silico analysis of functional and structural impact SNPS in the *MC1R* gene. J An Vet Adv. 2011;10: 928–931.

[71] Sturm RA, Teasdale RD, Box NF. Human pigmentation genes: identification, structure and consequences of polymorphic variation. Gene. 2001;277: 49–62. 11602344 10.1016/s0378-1119(01)00694-1

[72] Tully G. Genotype versus phenotype: Human pigmentation. Forensic Sci Int Genet. 2007;1: 105–110. 10.1016/j.fsigen.2007.01.005 19083738

[73] Nakayama K, Soemantri A, Jin F, Dashnyam B, Ohtsuka R, Duanchang P, et al Identification of novel functional variants of the melanocortin 1 receptor gene originated from Asians. Hum Genet. 2006;119: 322–330. 16463023 10.1007/s00439-006-0141-1

[74] Jiménez-Cervantes C, Germer S, González P, Sánchez J, Sánchez CO, García-Borrón JC. Thr40 and Met122 are new partial loss-of-function natural mutations of the human melanocortin 1 receptor. FEBS Lett. 2001;508: 44–48. 11707265 10.1016/s0014-5793(01)03025-3

[75] Sánches Más J, Olivares Sánchez C, Ghanem G, Haycock J, Teruel JAL, García-Borrón JC, et al Loss-of-function variants of the human melanocortin-1 receptor gene in melanoma cells define structural determinants of receptor function. Eur J Biochem. 2002;269: 6133–6141. 12473109 10.1046/j.1432-1033.2002.03329.x

