# Supplementary material for: Prediction of the Damage-Associated Non-Synonymous Single Nucleotide Polymorphisms in the Human MC1R Gene
Source: PLoS One. 2015 Mar 20;10(3):e0121812. doi: 10.1371/journal.pone.0121812 (PMC4368538; doi:10.1371/journal.pone.0121812)
Supplement: S1 Table — Results of the eleven individual tools, of the two consensus tools PON-P and PredictSNP 1.0. The nsSNPs in bold were selected by filter analysis. (DOC) [file pone.0121812.s001.doc]

# Supporting Information

**S1 Table: Prediction results of the nsSNPs in MC1R human gene.** Results of the eleven individual tools and of the two consensus tools PON-P and PredictSNP 1.0. The nsSNPs in bold were selected as tho most damaging by the all tools

| SNP ID | Mutation | SIFT | MutPred | PolyPhen-2 | PROVEAN | PANTHER | I-Mutant 3.0 | SNPs3D | Mutation assessor | PhD-SNP | SNPs&GO | SNAP | PON-P | PredictSNP 1.0 |
| --- | --- | --- | --- | --- | --- | --- | --- | --- | --- | --- | --- | --- | --- | --- |
| rs376929501 | M1I | Damaging | Deleterious | Probably damaging | Neutral | Tolerated | Neutral | Neutral | Neutral | Neutral | Neutral | Neutral | Unclassified | Deleterious |
| rs373990019 | G12D | Damaging | Neutral | Benign | Neutral | Tolerated | Disease | Neutral | Low | Disease | Neutral | Neutral | Neutral | Neutral |
| rs368247494 | L14F | Tolerated | Neutral | Benign | Neutral | Tolerated | Neutral | Neutral | Medium | Neutral | Neutral | Neutral | Neutral | Neutral |
| rs372041071 | T19I | Tolerated | Neutral | Benign | Neutral | Tolerated | Neutral | Neutral | Low | Neutral | Neutral | Neutral | Neutral | Neutral |
| rs376679503 | R34W | Tolerated | Neutral | Benign | Neutral | Tolerated | Disease | Neutral | Neutral | Neutral | Neutral | Neutral | Neutral | Neutral |
| rs369016553 | R34Q | Tolerated | Neutral | Benign | Neutral | Tolerated | Disease | Neutral | Neutral | Neutral | Neutral | Neutral | Neutral | Neutral |
| rs200050206 | V38M | Damaging | Deleterious | Benign | Neutral | Tolerated | Disease | Neutral | Medium | Neutral | Neutral | Neutral | Neutral | Neutral |
| rs369674161 | S41C | Damaging | Deleterious | Benign | Neutral | Deleterious | Disease | Deleterious | Neutral | Disease | Neutral | Non-neutral | Unclassified | Neutral |
| rs61996344 | F45L | Damaging | Deleterious | Probably damaging | Deleterious | Tolerated | Disease | Deleterious | Medium | Disease | Neutral | Non-neutral | Neutral | Deleterious |
| rs371156858 | S47I | Damaging | Deleterious | Benign | Neutral | Tolerated | Disease | Neutral | Medium | Neutral | Neutral | Neutral | Neutral | Neutral |
| rs201787533 | **L48P** | Damaging | Deleterious | Probably damaging | Deleterious | Deleterious | Disease | Deleterious | Medium | Disease | Disease | Non-neutral | Pathogenic | Deleterious |
| rs117952179 | A57V | Tolerated | Neutral | Benign | Neutral | Tolerated | Disease | Neutral | Neutral | Neutral | Neutral | Neutral | Neutral | Neutral |
| rs201632257 | A57T | Damaging | Neutral | Benign | Neutral | Tolerated | Neutral | Neutral | Neutral | Neutral | Neutral | Neutral | Neutral | Neutral |
| rs1805005 | V60L | Damaging | Neutral | Probably damaging | Neutral | Tolerated | Neutral | Neutral | Low | Neutral | Neutral | Neutral | Neutral | Neutral |
| rs368501338 | A64T | Tolerated | Neutral | Benign | Neutral | Tolerated | Disease | Neutral | Medium | Neutral | Neutral | Neutral | Neutral | Neutral |
| rs372590533 | **R67W** | Damaging | Deleterious | Probably damaging | Deleterious | Deleterious | Disease | Deleterious | High | Disease | Disease | Non-neutral | Pathogenic | Deleterious |
| rs34090186 | R67Q | Damaging | Neutral | Possibly damaging | Neutral | Deleterious | Disease | Neutral | Medium | Disease | Disease | Non-neutral | Pathogenic | Deleterious |
| rs377122753 | **H70Y** | Damaging | Deleterious | Probably damaging | Deleterious | Deleterious | Disease | Deleterious | High | Disease | Disease | Non-neutral | Unclassified | Deleterious |
| rs377297107 | **P72L** | Damaging | Deleterious | Probably damaging | Deleterious | Deleterious | Disease | Deleterious | Medium | Disease | Disease | Non-neutral | Pathogenic | Deleterious |
| rs371458749 | M73V | Damaging | Deleterious | Benign | Deleterious | Tolerated | Disease | Deleterious | Medium | Disease | Neutral | Non-neutral | Pathogenic | Deleterious |
| rs200759505 | I77M | Damaging | Deleterious | Probably damaging | Neutral | Deleterious | Neutral | Neutral | Medium | Disease | Neutral | Non-neutral | Unclassified | Deleterious |
| rs34474212 | **S83P** | Damaging | Deleterious | Probably damaging | Deleterious | Deleterious | Disease | Deleterious | High | Disease | Disease | Non-neutral | Pathogenic | Deleterious |
| rs1805006 | D84E | Damaging | Neutral | Probably damaging | Deleterious | Tolerated | Neutral | Deleterious | Medium | Disease | Neutral | Non-neutral | Pathogenic | Deleterious |
| SNP ID continuation | Mutation | SIFT | MutPred | PolyPhen-2 | PROVEAN | PANTHER | I-Mutant 3.0 | SNPs3D | Mutation assessor | PhD-SNP | SNPs&GO | SNAP | PON-P | PredictSNP 1.0 |
| rs34540312 | G89R | Damaging | Neutral | Possibly damaging | Neutral | Tolerated | Disease | Deleterious | Low | Disease | Neutral | Non-neutral | Pathogenic | Deleterious |
| rs2228479 | V92M | Tolerated | Neutral | Benign | Neutral | Tolerated | Disease | Neutral | Low | Neutral | Neutral | Neutral | Neutral | Neutral |
| rs34158934 | T95M | Tolerated | Neutral | Possibly damaging | Deleterious | Tolerated | Disease | Deleterious | Low | Neutral | Neutral | Non-neutral | Neutral | Neutral |
| rs373341896 | I98V | Tolerated | Neutral | Benign | Neutral | Tolerated | Neutral | Neutral | Low | Neutral | Neutral | Neutral | Neutral | Neutral |
| rs192276807 | L99P | Damaging | Deleterious | Benign | Deleterious | Deleterious | Disease | Neutral | Low | Disease | Neutral | Non-neutral | Unclassified | Deleterious |
| rs2229617 | G104S | Damaging | Neutral | Benign | Deleterious | Tolerated | Disease | Neutral | Medium | Neutral | Neutral | Non-neutral | Neutral | Neutral |
| rs201489928 | A111V | Damaging | Neutral | Benign | Neutral | Tolerated | Disease | Neutral | Low | Neutral | Neutral | Neutral | Neutral | Neutral |
| rs374827260 | D117V | Damaging | Deleterious | Probably damaging | Deleterious | Deleterious | Disease | Deleterious | Medium | Disease | Disease | Neutral | Unclassified | Deleterious |
| rs33932559 | I120T | Damaging | Neutral | Possibly damaging | Deleterious | Deleterious | Disease | Neutral | Medium | Neutral | Neutral | Neutral | Unclassified | Neutral |
| rs200616835 | D121E | Damaging | Deleterious | Probably damaging | Deleterious | Tolerated | Disease | Deleterious | Medium | Disease | Neutral | Non-neutral | Unclassified | Deleterious |
| rs201192930 | V122M | Damaging | Deleterious | Benign | Neutral | Tolerated | Disease | Neutral | Low | Neutral | Neutral | Neutral | Neutral | Neutral |
| rs374235260 | M128T | Damaging | Deleterious | Benign | Deleterious | Tolerated | Disease | Neutral | Low | Disease | Neutral | Neutral | Unclassified | Deleterious |
| rs377025135 | S130F | Damaging | Deleterious | Possibly damaging | Deleterious | Deleterious | Disease | Neutral | Medium | Neutral | Disease | Non-neutral | Unclassified | Deleterious |
| rs370094672 | S131N | Damaging | Deleterious | Probably damaging | Deleterious | Deleterious | Disease | Deleterious | High | Disease | Neutral | Non-neutral | Unclassified | Deleterious |
| rs376709880 | V140M | Tolerated | Deleterious | Benign | Neutral | Deleterious | Disease | Neutral | Medium | Disease | Neutral | Neutral | Neutral | Neutral |
| rs11547464 | R142H | Damaging | Neutral | Probably damaging | Deleterious | Deleterious | Disease | Deleterious | High | Disease | Disease | Non-neutral | Pathogenic | Deleterious |
| rs374423188 | A149T | Damaging | Deleterious | Probably damaging | Deleterious | Deleterious | Disease | Deleterious | High | Neutral | Neutral | Non-neutral | Unclassified | Deleterious |
| rs149922657 | **R151H** | Damaging | Deleterious | Probably damaging | Deleterious | Deleterious | Disease | Deleterious | Medium | Disease | Disease | Non-neutral | Unclassified | Deleterious |
| rs1805007 | R151G | Damaging | Neutral | Probably damaging | Deleterious | Deleterious | Disease | Deleterious | High | Disease | Disease | Non-neutral | Unclassified | Deleterious |
| rs1805007 | R151C | Damaging | Neutral | Probably damaging | Deleterious | Deleterious | Disease | Deleterious | High | Disease | Disease | Non-neutral | Pathogenic | Deleterious |
| rs1110400 | I155T | Damaging | Neutral | Probably damaging | Deleterious | Deleterious | Disease | Deleterious | Medium | Disease | Neutral | Non-neutral | Pathogenic | Deleterious |
| rs3212365 | V156L | Damaging | Neutral | Possibly damaging | Neutral | Tolerated | Neutral | Neutral | Low | Neutral | Neutral | Neutral | Unclassified | Neutral |
| rs201975178 | V156A | Damaging | Deleterious | Possibly damaging | Deleterious | Tolerated | Disease | Deleterious | Medium | Disease | Neutral | Non-neutral | Pathogenic | Deleterious |
| SNP ID Continuation | Mutation | SIFT | MutPred | PolyPhen-2 | PROVEAN | PANTHER | I-Mutant 3.0 | SNPs3D | Mutation assessor | PhD-SNP | SNPs&GO | SNAP | PON-P | PredictSNP 1.0 |
| rs104894524 | T157I | Damaging | Deleterious | Probably damaging | Deleterious | Tolerated | Disease | Deleterious | High | Disease | Neutral | Non-neutral | Pathogenic | Deleterious |
| rs104894523 | P159T | Damaging | Neutral | Benign | Neutral | Tolerated | Disease | Neutral | Low | Disease | Neutral | Non-neutral | Unclassified | Neutral |
| rs1805008 | R160W | Damaging | Neutral | Probably damaging | Deleterious | Deleterious | Disease | Deleterious | Medium | Disease | Disease | Non-neutral | Pathogenic | Deleterious |
| rs885479 | R163Q | Tolerated | Neutral | Benign | Neutral | Tolerated | Disease | Neutral | Medium | Neutral | Neutral | Non-neutral | Neutral | Neutral |
| rs35040147 | A166G | Damaging | Neutral | Benign | Neutral | Tolerated | Disease | Neutral | Low | Neutral | Neutral | Neutral | Neutral | Neutral |
| rs35784916 | A171S | Damaging | Neutral | Benign | Neutral | Tolerated | Disease | Neutral | Low | Neutral | Neutral | Neutral | Neutral | Neutral |
| rs373224783 | A171G | Tolerated | Neutral | Benign | Deleterious | Tolerated | Disease | Neutral | Low | Neutral | Neutral | Non-neutral | Neutral | Neutral |
| rs376670171 | **S172I** | Damaging | Deleterious | Probably damaging | Deleterious | Deleterious | Disease | Deleterious | High | Disease | Disease | Non-neutral | Unclassified | Deleterious |
| rs373044118 | Y183D | Damaging | Deleterious | Possibly damaging | Deleterious | Deleterious | Disease | Deleterious | Low | Disease | Disease | Non-neutral | Pathogenic | Deleterious |
| rs377580634 | F195V | Damaging | Deleterious | Possibly damaging | Deleterious | Tolerated | Neutral | Neutral | Medium | Disease | Neutral | Non-neutral | Unclassified | Deleterious |
| rs3212366 | F196L | Damaging | Neutral | Probably damaging | Deleterious | Deleterious | Neutral | Deleterious | Medium | Disease | Neutral | Non-neutral | Unclassified | Deleterious |
| rs374355873 | M203V | Damaging | Deleterious | Possibly damaging | Deleterious | Tolerated | Disease | Deleterious | Medium | Disease | Neutral | Non-neutral | Unclassified | Deleterious |
| rs377499038 | **L206P** | Damaging | Deleterious | Probably damaging | Deleterious | Deleterious | Disease | Deleterious | High | Disease | Disease | Non-neutral | Pathogenic | Deleterious |
| rs188462456 | L211V | Damaging | Deleterious | Possibly damaging | Neutral | Tolerated | Neutral | Deleterious | Medium | Neutral | Neutral | Non-neutral | Neutral | Deleterious |
| rs200000734 | R213W | Damaging | Deleterious | Benign | Neutral | Deleterious | Disease | Deleterious | Medium | Disease | Neutral | Non-neutral | Unclassified | Deleterious |
| rs200965363 | A218T | Damaging | Neutral | Possibly damaging | Neutral | Tolerated | Disease | Neutral | Medium | Neutral | Neutral | Neutral | Neutral | Neutral |
| rs371906898 | I221T | Damaging | Deleterious | Probably damaging | Deleterious | Deleterious | Disease | Deleterious | Medium | Disease | Neutral | Non-neutral | Unclassified | Deleterious |
| rs369807854 | A222T | Tolerated | Deleterious | Benign | Neutral | Tolerated | Neutral | Neutral | Medium | Neutral | Neutral | Neutral | Neutral | Neutral |
| rs374547608 | A222D | Damaging | Deleterious | Possibly damaging | Deleterious | Deleterious | Disease | Deleterious | Medium | Disease | Neutral | Non-neutral | Pathogenic | Deleterious |
| rs372152373 | R223G | Damaging | Deleterious | Benign | Neutral | Tolerated | Disease | Neutral | Medium | Disease | Neutral | Non-neutral | Unclassified | Deleterious |
| rs376780075 | R229H | Tolerated | Neutral | Benign | Neutral | Tolerated | Disease | Neutral | Neutral | Disease | Neutral | Neutral | Neutral | Neutral |
| rs368714912 | P230L | Tolerated | Deleterious | Benign | Neutral | Deleterious | Disease | Neutral | Medium | Neutral | Neutral | Neutral | Neutral | Neutral |
| rs200051702 | **T242I** | Damaging | Deleterious | Probably damaging | Deleterious | Deleterious | Disease | Deleterious | High | Disease | Disease | Non-neutral | Unclassified | Deleterious |
| SNP ID Continuation | Mutation | SIFT | MutPred | PolyPhen-2 | PROVEAN | PANTHER | I-Mutant 3.0 | SNPs3D | Mutation assessor | PhD-SNP | SNPs&GO | SNAP | PON-P | PredictSNP 1.0 |
| rs201028944 | L252F | Damaging | Deleterious | Benign | Neutral | Tolerated | Disease | Neutral | Low | Neutral | Neutral | Neutral | Neutral | Neutral |
| rs371214731 | **G255R** | Damaging | Deleterious | Probably damaging | Deleterious | Deleterious | Disease | Deleterious | Medium | Disease | Disease | Non-neutral | Pathogenic | Deleterious |
| rs200215218 | **P256S** | Damaging | Deleterious | Probably damaging | Deleterious | Deleterious | Disease | Deleterious | High | Disease | Disease | Non-neutral | Unclassified | Deleterious |
| rs376508354 | F257Y | Damaging | Deleterious | Probably damaging | Deleterious | Deleterious | Neutral | Neutral | Medium | Disease | Neutral | Non-neutral | Unclassified | Deleterious |
| rs370073863 | L263F | Damaging | Deleterious | Possibly damaging | Deleterious | Deleterious | Disease | Deleterious | Medium | Disease | Neutral | Neutral | Unclassified | Deleterious |
| rs371583010 | E269G | Damaging | Neutral | Benign | Neutral | Tolerated | Disease | Neutral | Neutral | Disease | Neutral | Neutral | Unclassified | Neutral |
| rs12102534 | T272M | Damaging | Deleterious | Probably damaging | Neutral | Tolerated | Disease | Deleterious | Medium | Neutral | Neutral | Non-neutral | Neutral | Deleterious |
| rs368281517 | **C273Y** | Damaging | Deleterious | Probably damaging | Deleterious | Deleterious | Disease | Deleterious | High | Disease | Disease | Non-neutral | Unclassified | Deleterious |
| rs201171524 | K278E | Damaging | Deleterious | Possibly damaging | Neutral | Tolerated | Disease | Neutral | Low | Neutral | Neutral | Non-neutral | Unclassified | Deleterious |
| rs376692024 | N279S | Damaging | Deleterious | Benign | Neutral | Tolerated | Disease | Neutral | Medium | Neutral | Neutral | Non-neutral | Neutral | Neutral |
| rs202197434 | N279K | Damaging | Deleterious | Probably damaging | Neutral | Tolerated | Disease | Deleterious | Medium | Disease | Neutral | Neutral | Neutral | Deleterious |
| rs141177570 | N281S | Damaging | Deleterious | Benign | Deleterious | Tolerated | Disease | Deleterious | Low | Disease | Neutral | Non-neutral | Unclassified | Deleterious |
| rs373957223 | I287M | Damaging | Deleterious | Probably damaging | Neutral | Deleterious | Neutral | Deleterious | Medium | Neutral | Neutral | Non-neutral | Neutral | Deleterious |
| rs369542041 | **C289R** | Damaging | Deleterious | Probably damaging | Deleterious | Deleterious | Disease | Deleterious | Medium | Disease | Disease | Non-neutral | Pathogenic | Deleterious |
| rs373703770 | N290S | Tolerated | Deleterious | Benign | Deleterious | Tolerated | Disease | Deleterious | Medium | Disease | Neutral | Non-neutral | Neutral | Deleterious |
| rs376425491 | I292T | Damaging | Neutral | Benign | Deleterious | Tolerated | Disease | Neutral | Low | Neutral | Neutral | Non-neutral | Neutral | Deleterious |
| rs1805009 | D294H | Damaging | Neutral | Probably damaging | Deleterious | Deleterious | Disease | Deleterious | Medium | Disease | Disease | Non-neutral | Pathogenic | Deleterious |
| rs373467427 | I297V | Damaging | Deleterious | Benign | Neutral | Deleterious | Neutral | Deleterious | Medium | Neutral | Neutral | Non-neutral | Unclassified | Neutral |
| rs370472871 | A299T | Damaging | Deleterious | Probably damaging | Deleterious | Deleterious | Neutral | Deleterious | Medium | Disease | Neutral | Neutral | Unclassified | Deleterious |
| rs373872609 | F300C | Damaging | Deleterious | Probably damaging | Deleterious | Deleterious | Disease | Neutral | Medium | Disease | Neutral | Non-neutral | Unclassified | Deleterious |
| rs368507952 | **R306H** | Damaging | Deleterious | Probably damaging | Deleterious | Deleterious | Disease | Deleterious | High | Disease | Disease | Non-neutral | Unclassified | Deleterious |
| rs375127718 | T308M | Damaging | Deleterious | Probably damaging | Deleterious | Deleterious | Neutral | Deleterious | Medium | Disease | Neutral | Non-neutral | Neutral | Deleterious |
| rs377248188 | C315S | Damaging | Deleterious | Benign | Deleterious | Tolerated | Neutral | Deleterious | Medium | Disease | Disease | Non-neutral | Unclassified | Deleterious |
