# Supplementary material for: Prediction of the Damage-Associated Non-Synonymous Single Nucleotide Polymorphisms in the Human MC1R Gene
Source: PLoS One. 2015 Mar 20;10(3):e0121812. doi: 10.1371/journal.pone.0121812 (PMC4368538; doi:10.1371/journal.pone.0121812)
Supplement: S2 Table — The data in dbSNP (NCBI) and Uniprot databases about the nsSNPs classified as pathogenic and the alleles associated with RHC phenotype in literature. R: alleles with high penetrance; r: alleles with low penetrance in RHC. * alleles with divergences in the RHC classification. (DOC) [file pone.0121812.s002.doc]

# Supporting Information

**S2 Table: Information available about the MC1R nsSNPs.**

| SNP ID | Mutation | dbSNP | Uniprot database | RHC | Damage associated results | Consensus Prediction | Reference |
| --- | --- | --- | --- | --- | --- | --- | --- |
| rs200050206 | V38M | NA | Moderate decrease in coupling to the cAMP pathway; reduced cell surface expression as a consequence of retention in the endoplasmic reticulum | - | 3 | Neutral | 8 |
| rs1805005 | V60L | Pathogenic | Associated with a risk for developing melanoma; unable to stimulate cAMP production as strongly as the wild type receptor in response to alpha melanocyte-stimulating hormone stimulation. | r | 2 | Neutral | 7 |
| rs34090186 | R67Q | NA | Shows a moderate and not significant decrease of cAMP production to NDP-MSH stimulation; shows a decreased responses to low concentrations of NDP- MSH stimulation | - | 8 | Deleterious | 73 |
| rs1805006 | D84E | Risk factor | May be associated with a risk for developing cutaneous malignant melanoma (CMM5); | R | 7 | Deleterious | 67 |
| rs2228479 | V92M | Pathogenic | Associated with a risk for developing melanoma; predominantly found in type I skin; shows a moderate and not significant decreased of cAMP production to NDP-MSH stimulation | r | 1 | Neutral | 67 |
| rs33932559 | I120T | NA | Shows a moderate and not significant decrease of cAMP production to NDP-MSH stimulation; shows decreased responses to low concentrations of NDPMSH stimulation | - | 6 | Neutral | 73 |
| rs201192930 | V122M | NA | Associated with fair hair and light skin; loss-of-function | - | 3 | Neutral | 74 |
| rs374235260 | M128T | NA | Susceptibility to cutaneous malignant melanoma (CMM5); Complete absence of functional coupling to the cAMP pathway; trafficked to the cell surface but unable to bind agonist efficiently | - | 5 | Neutral | 8 |
| SNP ID continuation | Mutation | dbSNP | Uniprot database | RHC | Damage associated results | Consensus Prediction | Reference |
| rs11547464 | R142H | NA | NA | R* | 10 | Deleterious | 75 |
| rs1805007 | R151C | Pathogenic | Associated with red hair and light skin of type I; binds to α-MSH but cannot be stimulated to produce cAMP | R | 10 | Deleterious | 62 |
| rs1110400 | I155T | NA | Associated with a risk for developing melanoma | r* | 9 | Deleterious | 7 |
| rs104894524 | T157I | Risk factor | Associated with UV induced susceptibility to skin damage; shows a dramatically decreased cAMP production to NDP-MSH stimulation | - | 9 | Deleterious | 73 |
| rs104894523 | P159T | Risk factor | Associated with UV induced susceptibility to skin damage; shows a strong decreased cAMP production to NDP- MSH stimulation | - | 4 | Neutral | 73 |
| rs1805008 | R160W | Pathogenic | Associated with a risk for developing melanoma; unable to stimulate cAMP production as strongly as the wild type receptor in response to α-MSH stimulation | R | 10 | Deleterious | 7 |
| rs885479 | R163Q | NA | Associated with a risk for developing melanoma; shows a moderate and not significant decrease of cAMP production to NDP-MSH stimulation; shows a not significant decrease in cAMP production at any concentrations of NDP- MSH stimulation | r | 3 | Neutral | 7 |
| rs35040147 | A166G | NA | Shows a moderate and not significant decrease of cAMP production to NDP-MSH stimulation; shows a not significant decrease in cAMP production at any concentrations of NDP-MSH stimulation | - | 2 | Neutral | 73 |
| rs141177570 | N281S | NA | Functionally silent polymorphism not affecting receptor surface expression | - | 7 | Deleterious | 8 |
| rs369542041 | C289R | NA | Susceptibility to cutaneous malignant melanoma (CMM5); Complete absence of functional coupling to the cAMP pathway; trafficked to the cell surface but unable to bind agonist efficiently | - | 11 | Deleterious | 8 |
| SNP ID | Mutation | dbSNP | Uniprot database | RHC | Damage associated results | Consensus Prediction | Reference |
| rs1805009 | D294H | Pathogenic | Associated with a risk for developing melanoma; unable to stimulate cAMP production as strongly as the wild type receptor in response to alpha-melanocyte-stimulating hormone stimulation | R | 10 | Deleterious | 7 |

The data in dbSNP (NCBI) and Uniprot databases about the nsSNPs classified as pathogenic and the alleles associated with RHC phenotype in literature. R: alleles with high penetrance; r: alleles with low penetrance in RHC. * alleles with divergences in the RHC classification.
